# Does Intensive Glucose Control Prevent Cognitive Decline in Diabetes? A Meta-Analysis

**DOI:** 10.1155/2015/680104

**Published:** 2015-08-04

**Authors:** Carlos Peñaherrera-Oviedo, Daniel Moreno-Zambrano, Michael Palacios, María Carolina Duarte-Martinez, Carlos Cevallos, Ximena Gamboa, María Beatriz Jurado, Leonardo Tamariz, Ana Palacio, Rocío Santibañez

**Affiliations:** ^1^Universidad Catolica de Santiago de Guayaquil, 090112 Guayas, Ecuador; ^2^Miller School of Medicine, University of Miami, Miami, FL 33136, USA

## Abstract

Diabetes mellitus is associated with cognitive decline and impaired performance in cognitive function tests among type 1 and type 2 diabetics. Even though the use of tight glucose control has been limited by a reported higher mortality, few reports have assessed the impact of treatment intensity on cognitive function. We conducted a meta-analysis to evaluate if an intensive glucose control in diabetes improves cognitive function, in comparison to standard therapy. We included 7 studies that included type 1 or type 2 diabetics and used standardized tests to evaluate various cognitive function domains. Standardized mean differences (SMDs) were calculated for each domain. We found that type 1 diabetics get no cognitive benefit from a tight glucose control, whereas type 2 diabetics get some benefit on processing speed and executive domains but had worse performances in the memory and attention domains, along with a higher incidence of mortality when using intensive glucose control regimes.

## 1. Introduction

Diabetes mellitus (DM) is a chronic metabolic condition that affects 8.3% of the world population and causes significant morbidity and mortality. The number of people suffering from diabetes is expected to increase beyond 592 million people over the next 25 years [1, 2]. Endothelial damage in diabetes leads to damage of multiple organs and an increased risk of myocardial infarction, stroke, and peripheral vascular disease, along with other chronic complications such as kidney disease or retinopathy [1]. Diabetes also increases the risk of cognitive dysfunction and both vascular dementia and Alzheimer's disease [3–5]. This association is more prominent in elderly diabetics, although mild cognitive impairment may be present also in relatively younger diabetics [6–8]. The impact of diabetes in cognitive function may become more apparent as the life expectancy has significantly increased over the past years [2].

A recent meta-analysis determined that type 2 diabetics had worse performance in neuropsychological tests when compared to normal subjects [9]. As for type 1 diabetes, which is less common and has an onset in childhood, information relating to cognitive function is relatively scarce [1]. There is, however, evidence of an overall decrease in pediatric cognitive performance for diabetic children except in the memory and language domains [10]. A more recent study showed a nonstatistically significant reduction of intellectual function for type 1 diabetics when compared to normal children [11].

Although recent data has found that intensive glucose control could be associated with increased mortality among diabetics, the impact on cognitive function is less understood [12]. We conducted a meta-analysis to determine if intensive glucose control can actually prevent or delay the onset of cognitive decline both in type 1 and in type 2 diabetics. As we move to achieve patient centered care, having information for patients regarding the balance between quantity and quality of life will be useful.

## 2. Materials and Methods

### 2.1. Search Strategy

PubMed (MEDLINE) database was searched for randomized controlled trials published from January 1, 1980, to June 1, 2014, using MeSH terms and keywords. Search terms used included “type 1 diabetes mellitus,” “type 2 diabetes mellitus,” “drug therapy,” and “cognitive function.” The full search including MeSH terms was (((diabetes mellitus, type 1/drug therapy [MeSH Terms] OR diabetes mellitus, type 2/drug therapy [MeSH Terms]) OR diabetes mellitus, type 1/therapy [MeSH Terms]) OR diabetes mellitus, type 2/therapy [MeSH Terms]) AND (cognitive [All Fields] AND (“physiology” [Subheading] OR “physiology” [All Fields] OR “function” [All Fields] OR “physiology” [MeSH Terms] OR “function” [All Fields])) AND ((Clinical Trial [ptyp] OR Randomized Controlled Trial [ptyp]) AND (“1980/01/01” [PDAT]: “2014/12/31” [PDAT])). We also reviewed the reference list of the identified articles looking for additional studies that might be included in this meta-analysis.

### 2.2. Inclusion Criteria

We included randomized controlled trials (RCT), which analyzed patients with either type 1 or type 2 diabetes, had at least one group of patients receiving intensive glucose control and another receiving conventional antidiabetic treatment, and provided information regarding assessment of cognitive function after a follow-up period using a standardized method.

### 2.3. Exclusion Criteria

The exclusion criteria we used were as follows: studies which included patients already diagnosed with cognitive dysfunction or established dementia, studies that used only the Minimental Score Examination (MMSE) as an assessment of cognitive function, and studies that utilized a cognitive testing method which was not comparable to those used in any of the other articles included.

### 2.4. Definition of the Exposure

We defined interventions as “intensive” if they tailored care to reach a glycated hemoglobin (HbA1c) goal of less than 7% or a fasting glucose level of less than 130 mg/dL. The format and content of the interventions could vary. Conventional treatment was defined simply as the continuation of the regular treatment the patient was already receiving.

The definition of intensive glucose control varied among the included studies. Four of them intended to achieve levels of HbA1c below 6%, while another one targeted HbA1c levels below 7% [14–16, 18, 19]. Two more studies did not report a goal level of glycated hemoglobin, one of them targeted preprandial glucose levels below 130 mg/dL instead, and the last one adjusted goals of glycaemia and HbA1c individually with each patient [13, 17]. Treatment goals are summarized in Table 1. The methods used to achieve these goals ranged from multifactorial behavioral interventions to adjusted doses of oral antidiabetics to 3 or more insulin injections per day or continuous insulin infusion with an external pump.

### 2.5. Outcome

The main outcome was cognitive dysfunction classified into the following domains based on standard domain definitions: information processing speed, executive function, attention/concentration, verbal memory, and motor function.

The domains were evaluated using validated neuropsychological tests. Information processing speed was assessed through the Digit-Symbol Substitution Subtest (DSST) of the Wechsler Assessment of Intelligence Scale (WAIS), in which the participant is initially shown a key containing symbol-digit pairs and must later copy the corresponding symbol under a series of numbers with empty boxes below [20]. Total score is given by the number of correct pairings within a 90-second limit. As measures of executive functioning, participants were assessed using the Trail Making Test part B (TMT-B) and the Similarities subtest of the WAIS. The TMT-B measures the time a subject needs to draw lines connecting 25 encircled letters and numbers distributed over a sheet of paper in alternating order [21]. For the Similarities subtest, subjects are asked in what way two words are alike (i.e., poem and statue). The scores for the Similarities task are presented in age-adjusted scaled scores.

Memory function was evaluated using the Rey Auditory Verbal Learning Test (RAVLT), a verbal learning task where the participant is given a list of 15 unrelated words repeated over 5 trials [22]. A delayed-recall trial is administered 30 minutes after the initial learning phase and the number of freely recalled words is recorded. Reaction time to auditory and visual stimuli was measured through computerized tasks where participants had to press a key immediately after presentation of visual (light) or auditory (tone) stimuli. The Finger Tapping Test was administered as a measure of motor function. In this test, participants place their hand on a board with a lever and tap their index finger on the lever as quickly as possible, using their dominant and nondominant hands, within a 10-second time interval. Scores are calculated by averaging the number of taps over five consecutive trials within a 5-point range with each hand [23]. The Stroop test is a measure of selective attention, cognitive flexibility, and cognitive inhibition [24]. It consists of three parts. In the first part subjects read a list of color names printed in black ink. In the second part they must name the color of a list of X's or color patches, depending on the version used. In the third part of the task the subject must name the color of a color word written in nonmatching ink color (e.g., the word green printed in red). A Stroop interference effect occurs when color-naming speed is significantly reduced as the subject must inhibit an automatic reading response to produce a more effortful color-naming response [25].

### 2.6. Statistical Analysis

We reported relevant baseline characteristics for each study as mean and percentage as reported. To aggregate unweighted results for all studies we report the median and interquartile range for continuous variables and for HbA1c we report the mean values before and after the intervention per randomized group. To assess for heterogeneity across studies we used the Cochran *Q* chi-square (significance level <0.10) and the *I*-squared statistic (>50%).

For the mathematical pooling we stratified the analysis by type of diabetes and calculated the standardized mean difference (SMD) with the corresponding 95% confidence interval and *p* value. The SMD represents the difference between the mean and standard deviation of the cognitive test in the intensive control group minus that of the conventional group for each study. The SMD was weighted by the sample size of each individual study per randomized group. We used Comprehensive Meta-Analysis software (Biostat Inc., Englewood, NJ) for the quantitative analysis.

## 3. Results

**Figure 1:**
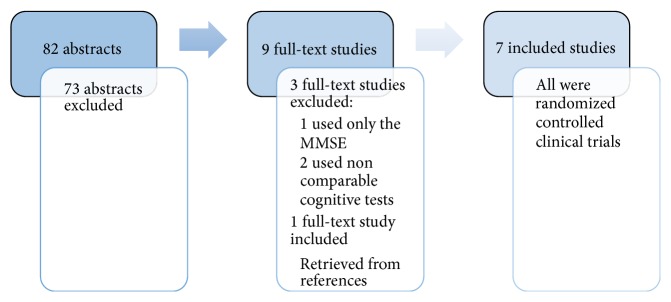
Summary of database search conducted on PubMed and details of study selection.

Our search strategy yielded 82 articles, from which we excluded 73 abstracts because they were not RCT or did not meet inclusion criteria. From the remaining 9 studies from the original search, we removed 3 more articles after exclusion criteria were applied. One additional study was retrieved from the references of the articles reviewed and was included for analysis as it did not meet exclusion criteria [16]. A total of 7 articles were finally included in the meta-analysis, of which 4 analyzed type 1 diabetics and 3 studied type 2 diabetics (Figure 1).

The combined sample size was 6056 patients (3011 under intensive glucose control and 3045 under conventional treatment). The median age was 27 years for type 1 diabetics and 62.4 years for type 2 diabetics. The median follow-up time was for type 1 and type 2. Only two studies had more than 50% female patients. Median baseline levels of HbA1c were 9.24% for the intensive treatment group and 9.07% for the conventional treatment patients, while HbA1c levels after treatment follow-up were 7.43% for the intensive group and 8.17% for the conventional treatment patients. Study characteristics are presented in Table 2.

**Figure 2:**
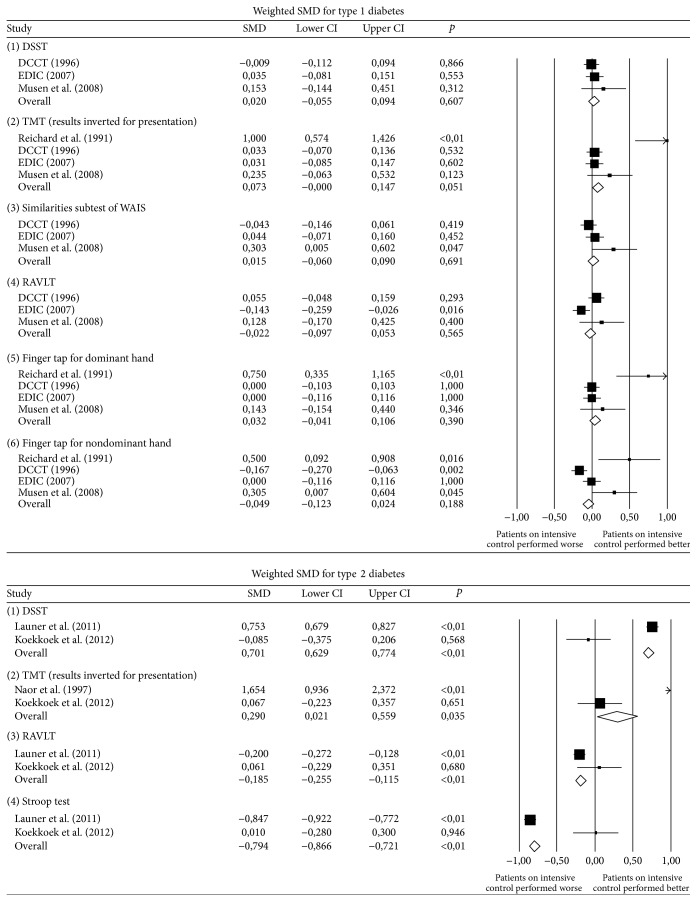
Summary of standardized mean differences for each cognitive test, divided by type of diabetes. Results for TMT have been mirrored for a more uniform presentation.

Table 3 describes the results of each test per arm. The most commonly reported tests where the DSST, trail making, finger tap, and RAVLT. The univariate results show that on each test there is a difference between the intensive treatment group and the control group.  Table 4 shows the weighted SMDs of each test stratified by type of diabetes. All tests for type 1 diabetes were nonsignificant. For type 2 diabetes the DSST SMD had a positive direction (0.71), while the Trail Making Test, Stroop test, and RAVLT had a negative direction. However, a negative direction on the SMD for Trail Making Test also favors intensive glucose control due to the nature of the test. These results are summarized in Figure 2, where results for TMT have been mirrored to a positive sense for a better presentation.

## 4. Discussion

Our meta-analysis demonstrates that tight glucose control is not superior to conventional care at preventing cognitive decline among type 1 diabetics and has a positive impact only on the information processing speed and executive functions among type 2 diabetics.

The lack of effect seen for type 1 diabetics could be related to the nonsignificant differences described to date in cognitive function between diabetics and healthy control groups [11]. Among young diabetic patients who are free of multiple comorbidities, the effect of hyperglycemia may not be severe enough to cause a significant cognitive impairment, and thus the glucose lowering regime used to treat diabetes becomes unimportant regarding prevention of cognitive function loss. An alternative explanation is that the small cognitive decline reported among type 1 diabetics is related to the effect of repeated hypoglycemic events which may cause white matter damage but would not be reduced by tight glucose control [10, 26].

In contrast, the effect of tight glucose control varied across cognitive domains among type 2 diabetics. The intensive control group performed significantly better on the DSST and TMT but did worse than the conventional treatment group on the Stroop test and the RAVLT. From these results we can conclude that tight glucose control favors the domains of information processing speed and executive function, but at the cost of negatively affecting attention and memory functions. The presence of comorbidities at the age of onset of diabetes, which is much later than that for type 1 diabetics, may help explain these results. Also, it has been described that insulin is one of the molecules that regulate tau protein phosphorylation in neurons, and thus insulin resistance may disrupt this process, causing tau to bind to microtubules, giving rise to the pathogenesis of Alzheimer's disease and dementia [27]. Educational level is also an important confounding factor in this population, as it has been observed that cognitive performance correlates directly with the amount of years of completed study [28]. However, there are no enough data to test the impact of these confounders in the current meta-analysis.

In regard to the higher risk of mortality previously reported for tight glucose control regimes, only two of the studies included reported a mortality outcome [15, 18]. Thus, evaluating the relationship between cognitive decline, mortality, and tight glucose control was not possible. To date, age, the increased risk of hypoglycemia, and the presence of important comorbidities are factors that favor the increased incidence of deaths in type 2 population, while in type 1 diabetics, though there was an increased risk of hypoglycemic events, the great majority were nonfatal [12, 15]. Further studies are needed to understand the relationship between cognitive decline and mortality.

Our study has several limitations. First, while there is significant evidence on the relationship of diabetes and cognitive decline, very few trials have addressed the impact of different glucose control regimes on cognitive function. More so, many studies evaluating this question could not be included because they either used noncomparable tests or reported cognitive decline using only the MMSE [26, 28–30]. The MMSE does not offer enough information to rigorously evaluate cognitive function. Also, the large variation in sample size among type 2 diabetes studies caused one of the studies to carry more significant weight than the others.

In conclusion, we observed that there is no benefit from intensive glucose lowering regarding cognitive function for the young type 1 diabetics, while the older type 2 diabetics benefit from this therapy in the domains of information processing speed and executive function but find their attention and memory hindered. These findings provide insight into the pathophysiology of different types of cognitive impairment and possible therapeutic avenues in the future. Some studies have shown an increased risk of cardiovascular mortality and hypoglycemia when using intensive glucose control regimes. Thus, each case should be evaluated individually to assess the benefits of a tight glycemic control against the observed risks. Since these complications are more common in older diabetic patients, intensive control of the glucose levels might be safer and more recommendable in type 1 diabetics, most of which are children or young adolescents, regarding noncognitive benefits.

## Figures and Tables

**Table 1:** Treatment goals for the definition of intensive glucose control.

Study	HbA1c (%)	Preprandial glucose level (mg/dL)
Reichard et al. [13]	Individual adjustment	Individual adjustment
DCCT [14]	<6.05	70–120
EDIC [15]	<6	70–120
Musen et al. [16]	<6.05	70–120
Naor et al. [17]	N/A	<130
Launer et al. [18]	<6	N/A
Koekkoek et al. [19]	<7	N/A

**Table 2:** Characteristics of included studies.

Study^*∗*^	Country	DM type	(*n*)	Intensive treatment (*n*)	Conventional treatment (*n*)	Mean (SD) age (years)		Follow-up time (months)	Female patients (%)	
						Intensive	Conventional		Intensive	Conventional
Reichard et al. [13]	Sweden	1	96	44	52	29.5 ± 1.1	31.6 ± 1	50	50	48
DCCT [14]	USA/Canada	1	1441	711	730	27.1 ± 7.1	26.5 ± 7.1	60	48.5	45.9
EDIC [15]	USA/Canada	1	1144	588	556	27 ± 7	27 ± 7	144	49	45
Musen et al. [16]	USA/Canada	1	175	82	93	16 ± 2	16 ± 2	144	50	62
Naor et al. [17]	Germany	2	40	20	20	63.6 ± 5.3	63.8 ± 5.5	2	60	65
Launer et al. [18]	USA/Canada	2	2977	1469	1508	62.3 ± 5.7	62.7 ± 5.9	40	48	49
Koekkoek et al. [19]	Netherlands	2	183	97	86	59.3 ± 5.6	59.5 ± 5.3	120	42.3	35.7

^*∗*^All included studies were randomized controlled clinical trials (RCCT).

DCCT: Diabetes Control and Complications Trial; EDIC: Epidemiology of Diabetes Interventions and Complications Study.

**Table 3:** Mean (SD) results from each cognitive function test utilized in the studies included.

Study	DM type	DSST (number of correct pairings in 50 seconds)		Trail Making Test (seconds)		Similarities subtest of WAIS (age-adjusted score 0–19)		Visual reaction time (milliseconds)		Auditory reaction time (milliseconds)		RAVLT (number of freely recalled words)		Stroop test (number of correct items in 45 seconds)		Finger tap for dominant hand (taps in 10 seconds)		Finger tap for nondominant hand (taps in 10 seconds)	
		I	C	I	C	I	C	I	C	I	C	I	C	I	C	I	C	I	C
Reichard et al. [13]^**∗**^	1	—	—	39.9	45.6	—	—	241	241	209	207	—	—	—	—	6.8	6.5	6.2	6.1

DCCT [14]	1	64.7 (11.3)	64.8 (11.2)	52.9 (16.7)	53.5 (19.6)	12.5 (2.4)	12.6 (2.3)	—	—	—	—	15.4 (1.7)	15.3 (1.9)	—	—	4.9 (0.7)	4.9 (0.7)	4.4 (0.6)	4.5 (0.6)

EDIC [15]	1	62.3 (11.4)	61.9 (11.4)	54.4 (20)	55 (18.8)	14 (2.2)	13.9 (2.3)	—	—	—	—	14.7 (2.2)	15 (2)	—	—	5.1 (0.7)	5.1 (0.8)	4.5 (0.7)	4.5 (0.7)

Musen et al. [16]	1	67.8 (10.5)	66.3 (9.1)	45.6 (12.8)	48.9 (15.1)	13.8 (2.2)	13.1 (2.4)	—	—	—	—	15.5 (1.4)	15.3 (1.7)	—	—	5.2 (0.7)	5.1 (0.7)	4.7 (0.6)	4.5 (0.7)

Naor et al. [17]	2	—	—	93.8 (17.1)	140 (35.6)	—	—	284.7 (22.8)	309.1 (54.7)	211.4 (42.8)	218.1 (38.1)	—	—	—	—	—	—	—	—

Launer et al. [18]	2	50.93 (0.43)	50.61 (0.42)	—	—	—	—	—	—	—	—	7.98 (0.1)	7.99 (0.09)	31.45 (0.36)	32.06 (0.36)	—	—	—	—

Koekkoek et al. [19]	2	55.6 (17.4)	57 (15.5)	93.5 (39.4)	96.5 (50.2)	—	—	8.3 (3.5)	8.1 (3)	—	—	8.3 (3.5)	8.1 (3)	50.2 (8.4)	50.1 (11.6)	—	—	—	—

^**∗**^Standard deviation (SD) not reported.

I: intensive glucose control group.

C: conventional therapy group.

“—” denotes that such test was not carried out in the corresponding study.

**Table 4:** Results of weighted SMDs for each cognitive test.

Cognitive test	Number of studies	Weighted SMD (95% CI)	*I*-squared	*p*
Type 1 diabetes				
DSST	3	0.02 (−0.05 to 0.09)	0%	0.60
Trail Making Test	4	−0.07 (−0.14 to 0.00)	85%	0.05
Similarities subtest of WAIS	3	0.015 (−0.06 to 0.09)	60%	0.69
RAVLT	3	−0.022 (−0.09 to 0.05)	72%	0.56
Finger tap from the dominant hand	4	0.032 (−0.04 to 0.106)	76%	0.39
Finger tap from nondominant hand	4	−0.045 (−0.123 to 0.024)	83%	0.19

Type 2 diabetes				
DSST	2	0.71 (0.64 to 0.78)	97%	<0.01
Trail Making Test	2	−0.29 (−0.55 to −0.02)	94%	0.04
RAVLT	2	−0.185 (−0.26 to −0.16)	66%	<0.01
Stroop test	2	−0.79 (−0.87 to −0.72)	97%	<0.01
